# Colonization by fragments of the submerged macrophyte *Myriophyllum spicatum* under different sediment type and density conditions

**DOI:** 10.1038/srep11821

**Published:** 2015-07-02

**Authors:** Feng Li, Lianlian Zhu, Yonghong Xie, Li Jiang, Xinsheng Chen, Zhengmiao Deng, Baihan Pan

**Affiliations:** 1 Key Laboratory of Agro-ecological Processes in Subtropical Region, The Chinese Academy of Sciences, Hunan 410125, China; 2 Dongting Lake Station for Wetland Ecosystem Research, Institute of Subtropical Agriculture, Changsha 410125, China; 3 Key Laboratory of Ecology of Rare and Endangered Species and Environmental Protection, Guangxi Normal University, Ministry of Education, 541004, China

## Abstract

In this paper, the effect of plant density, sediment type, and macrophyte fragment size on the fragment colonization ability of *Myriophyllum spicatum* was evaluated in an outdoor experiment. The relative growth rate (RGR) was higher in the mud and low-density treatments than in the sand and high-density treatments. The relative elongation rate (RER) decreased with increasing density and fragment size, with RER values being much higher in the mud than the sand treatments. Both branching number and shoot diameter increased with decreasing density and increasing fragment size, and were significantly higher in the mud than the sand treatments. The shoot : root ratio was higher in the mud treatments than in the sand treatments. Total N content in both the shoot and root was significantly higher in the mud and low-density treatments than in the sand and high-density treatments. Shoot P content only decreased with increasing density, while root P content was higher in the mud and low-density treatments than in the sand and high-density treatments. These data indicate that fragment colonization by *M. spicatum* is improved by large fragments, low density, and nutrient-rich sediments, and that these conditions contribute to the rapid population expansion of this species.

Vegetative fragments represent one of the most important mechanisms for the reproduction and spread of aquatic plants^1^. For instance, in Whalebone Cove Wetland (Connecticut, USA), 60% colonization events are completed by plant fragments^2^. These fragments usually descend to the sediment layer, where they produce roots, anchor, and establish new plants, contributing to plant dispersal and invasion^3^,^4^.

The success of fragment colonization strongly depends on the characteristics of a given species and several environment factors^5^,^6^. Disturbance with different intensities produces various types of fragments (e.g., different fragment lengths, with or without apices), which then significantly determine plant colonization fate. In general, larger fragments have greater colonization and regeneration abilities than smaller ones, due to their higher energy reserves and photosynthetic rates^7^,^8^. However, some studies have obtained different results^1^,^9^. Thus, the generality of the influence of fragment size on plant colonization ability requires confirmation.

Sediment nutrients are important for the growth of aquatic plants, providing a vital source of nutrients for plant growth^10^,^11^. In general, plants grown in sediments with high nutrient availability tend to have higher growth rates than those grown in sediments with lower nutrient availability^12^,^13^. However, the opposite results have also been obtained. For instance, the enrichment of sediment nutrients had no significant influence on the relative growth rate of *E. canadensis* and *C. cophocarpa*, which might be due to their low nutrient requirements^11^. In comparison, many studies have confirmed that sediment nutrients had a positive influence on the vegetation distribution of *Myriophyllum spicatum*^14^. Yet, the colonization of *M. spicatum* in Lake Wingra (Wisconsin, USA) is not correlated with the nutrient content of the substrate, with the plant growing over the entire littoral zone^15^. Thus, sediment type probably has a species-specific influence on plant colonization ability. Furthermore, plant responses might be influenced by both the nutrient content of the sediment and plant properties. The response of different sized fragments to the sediment might significantly differ due to differences in nutrient requirements. However, experimental evidence on this interactive influence is scarce.

Plant density represents another important biotic factor that determines fragment colonization and growth^13^. Higher plant densities are expected to inhibit plant growth, due to intensified competition for limited resources (e.g., light and nutrient). In contrast, when population densities are low, intraspecific facilitation may be important^16^,^17^. Moreover, the influence of plant density on plant growth might depend on resource availability and species characteristics. Competition intensity might be stronger when resources are scarce and individuals are larger in size. To date, the colonization success of fragments in relation to fragment size, water depth, and fragment position has been subject to detailed studies^1^,^6^,^7^,^9^. However, to our knowledge, no studies have focused on the combined effect of fragment size, plant density, and nutrient availability on plant colonization success.

*M. spicatum* is considered to be one of the most invasive aquatic weed species worldwide. This species forms thick monospecific stands that significantly reduce the biodiversity and abundance of various native aquatic plants^18^. *M. spicatum* exhibits three mechanisms of propagation: seed, rhizome production, and fragmentation. Vegetative dispersal by fragmentation represents the most important mechanism for population expansion by this species^19^. This study aimed to elucidate the interactive influence of fragment size, plant density, and nutrient availability on the colonization of *M. spicatum*. Specifically, two fragment sizes of *M. spicatum* were grown at two densities under two sediment types. Subsequently, the morphological (relative growth rate [RGR], relative elongation rate [RER], branching number, shoot diameter and biomass allocation and physiological characteristics (total N content and total P content in the shoot and root) were investigated. We hypothesize that *M. spicatum* performance increases when plant fragments are large, the sediment contains more nutrients, and the plant density is low. Following this general hypothesis, we predict that, (1) the RGR, RER, branching number, and shoot diameter of *M. spicatum* grown with longer fragment, lower density, and mud sediment conditions would be higher than that in plants grown with shorter fragment, higher density, and sand sediment conditions; (2) the shoot:root ratio of *M. spicatum* grown with longer fragment, lower density, and mud sediment conditions would be higher than that in plants with shorter fragment, higher density, and sand sediment conditions; and (3) total N content and total P content in the shoot and root would be higher for *M. spicatum* with longer fragment, lower density, and mud sediment conditions than that in plants with shorter fragment, higher density, and sand sediment conditions.

## Materials and Methods

### Plant materials

The shoots of *M. spicatum* were collected from a pond (N: 28°10'49.46'', E: 113°04'47.01'') at Hunan Agricultural University, Changsha, China, in April 2014. After collection, the shoots were placed in three water-filled buckets (22 L), and then transported to an experimental field at Dongting Lake Station for Wetland Ecosystem Research, the Chinese Academy of Sciences, China. The *M. spicatum* shoots were pre-incubated in tap water (containing 0.511 μg L^−1^ NH_4_^+^-N, 1.760 μg L^−1^ NO_3_^—^N, and 0.527 μg L^−1^ PO_4_^3+^-P, pH 7.2) for 15 days with natural sunshine.

### Experimental design

The experiment included two fragment sizes (6 cm and 12 cm), two sediment types (sand and mud), and two densities (88 plants m^−2^ and 704 plant m^−2^). Therefore, there were eight treatments in total: four low-density treatments (one plant per sediment type and per fragment size) and four high-density treatments (eight plants per sediment type and per fragment size). The density was designed according to the plant density in the pond, where average density is 390 ± 23 plants m^−2^.

After pre-incubation, a total of 540 apical shoots of *M. spicatum* (270 apical shoots of 6 cm and 12 cm, respectively) were planted in sand or mud-filled plastic pots (12 cm diameter, 15 cm height), with the lower 2 cm of the shoots being buried in the sediment. Mud was collected from Dongting Lake, and contained 2.02% organic matter, 19.49 μg g^−1^ total N, and 0.77 μg g^−1^ total P. Sand was collected from a local river (Xiang River), and contained 0.39% organic matter, 1.30 μg g^−1^ total N, and 0.30 μg g^−1^ total P. A total of 24 pots (3 pots per treatment) were placed into 5 larger plastic bins (98 cm × 76 cm × 68 cm) to control water level. The plastic bins were placed in an outdoor area with natural sunshine. The initial water level was maintained at 20 cm. After 7 days, the water level was raised to 50 cm with tap water (containing 0.511 *μ*g L^−1^ NH_4_^+^-N, 1.760 *μ*g L^−1^ NO_3_^−^-N and 0.527 *μ*g L^−1^ PO_4_^3+^-P, pH = 7.2), and surplus water was removed after rainfall to control water level. We also replenished the water once a week to prevent algal growth. Prior to the experiment, the biomass accumulation of 10 shoots from each fragment size was measured to calculate the relative growth rate of the plants.

### Harvest and Measurement

Plants that had formed a dense canopy in each bin were harvested after 17 weeks treatment. The incubation time was designed based on one of our previous studies. Plant roots were carefully dug out and cleaned with tap water. For each plant, shoot diameter was measured with a Vernier caliper and branching number was counted. Subsequently, the plants were divided into shoots (leaves and stems) and roots, oven dried at 80 °C for 48 h, and weighed. RGR and RER were calculated using the following equation: RGR = (lnw_2_–lnw_1_) / (t_2_–t_1_) and RER = (lnh_2_–lnh_1_) / (t_2_–t_1_), respectively, where w_1_ is the initial dry mass, w_2_ is the dry mass at harvest time t_2_, h_1_ is the initial height, h_2_ is the height at harvest time t_2_, and (t_2_–t_1_) is the experimental time. The shoot : root ratio was determined as the ratio of shoot dry mass to root dry biomass.

### Plant N and P content

The plant parts were ground into powder, and mixed together to measure plant chemistry. All samples were digested with H_2_SO_4_–H_2_O_2_, and analyzed for plant N and P content using colorimetric analysis on a TU-1901 spectrophotometer^20^,^21^. Three replicates were used to determine plant N and P content.

### Statistical analysis

A three-way ANOVA was performed, with fragment size, sediment type, and density as the main factors, to determine the effect of these factors on RGR, RER, shoot diameter, branching number, biomass allocation, total N content, and total P content of *M. spicatum* shoots and roots. Multiple comparisons of means were performed using Duncan’s test at the 0.05 significance level. Data were log_10_-transformed if necessary, to reduce heterogeneity of variance. Normality and homogeneity were tested using Lilliefors and Levene’s tests, respectively. All analyses were performed using the software SPSS 15.0 for Windows.

## Results

### RGR and RER

**Figure 1:**
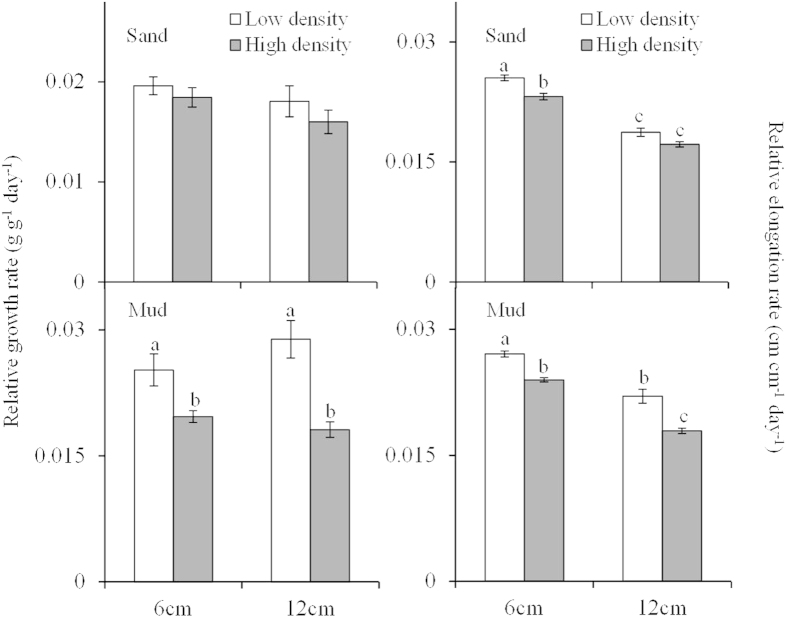
Relative growth rate (means ± SE, n = 5) and relative elongation rate (means ± SE, n = 5) of *Myriophyllum spicatum* with different fragment size growing in two densities and two sediment types.

The RGR of *M. spicatum* was significantly influenced by plant density and sediment type, rather than fragment size (Table 1; Fig. 1), and decreased with increasing plant density in the mud treatment. In contrast, plant density had an insignificant effect on RGR in the sand treatment. RGR was much higher in the mud treatment compared to the sand treatment. The highest RGR occurred in the low plant density +12 cm fragment + mud treatment (0.029 ± 0.00 g g^−1^ day^−1^). This value was 1.80 times higher than the lowest RGR, which occurred in the high plant density + 12 cm fragment + sand treatment (0.016 ± 0.00 g g^−1^ day^−1^; Fig. 1).

Sediment type, plant density, and fragment size all had a significant influence on the RER of *M. spicatum* (Table 1; Fig. 1), all of which decreased significantly with increasing plant density and fragment size. Moreover, the RER was much higher in the mud treatments compared to the sand treatments (Table 1; Fig. 1).

### Branching number and shoot diameter

**Figure 2:**
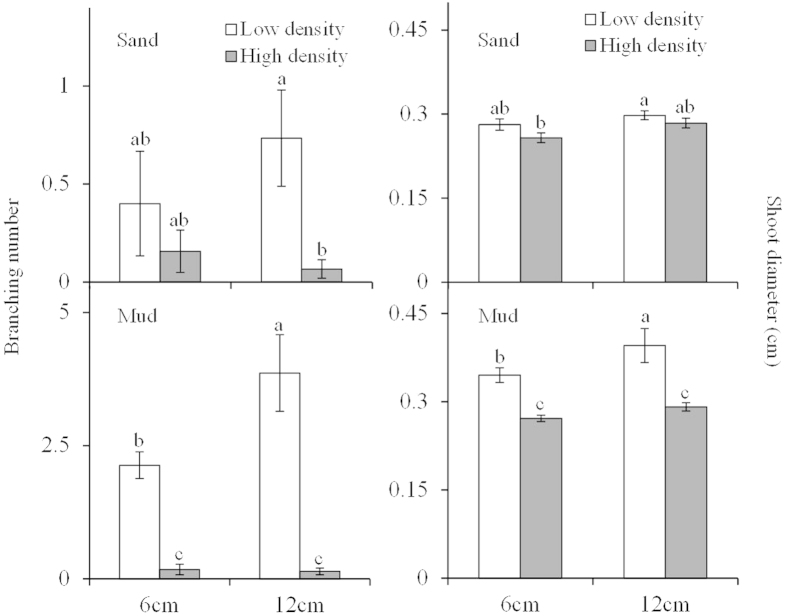
Branching number (means ± SE, n = 5) and shoot diameter (means ± SE, n = 5) of *Myriophyllum spicatum* with different fragment size growing in two densities and two sediment types.

Branching number differed significantly with fragment size, plant density, and sediment type (Table 1; Fig. 2), and was greater in the mud treatments compared to the sand treatments. In the mud treatments, branching number increased with decreasing plant density and increasing fragment size. The highest branching number occurred in the low density +12 cm fragment + mud treatment (3.87 ± 0.72; Fig. 2), and was 58 times higher than the lowest branching number in the high density +12 cm fragment + sand treatment (0.07 ± 0.05; Fig. 2).

Shoot diameter was significantly affected by sediment type, plant density, and fragment size (Table 1; Fig. 2), with shoot diameter increasing with decreasing plant density and increasing fragment size, especially in the mud treatments. At the same fragment size and plant density treatments, shoot diameter was much larger in the mud treatments compared to the sand treatments. Low density +12 cm fragment + mud treatment produced the highest shoot diameter (0.40 ± 0.03 cm; Fig. 2), which was 1.54 times greater than the lowest shoot diameter obtained from the high density + 6 cm fragment + sand treatment (0.26 ± 0.01 cm; Fig. 2).

### Biomass allocation

**Figure 3:**
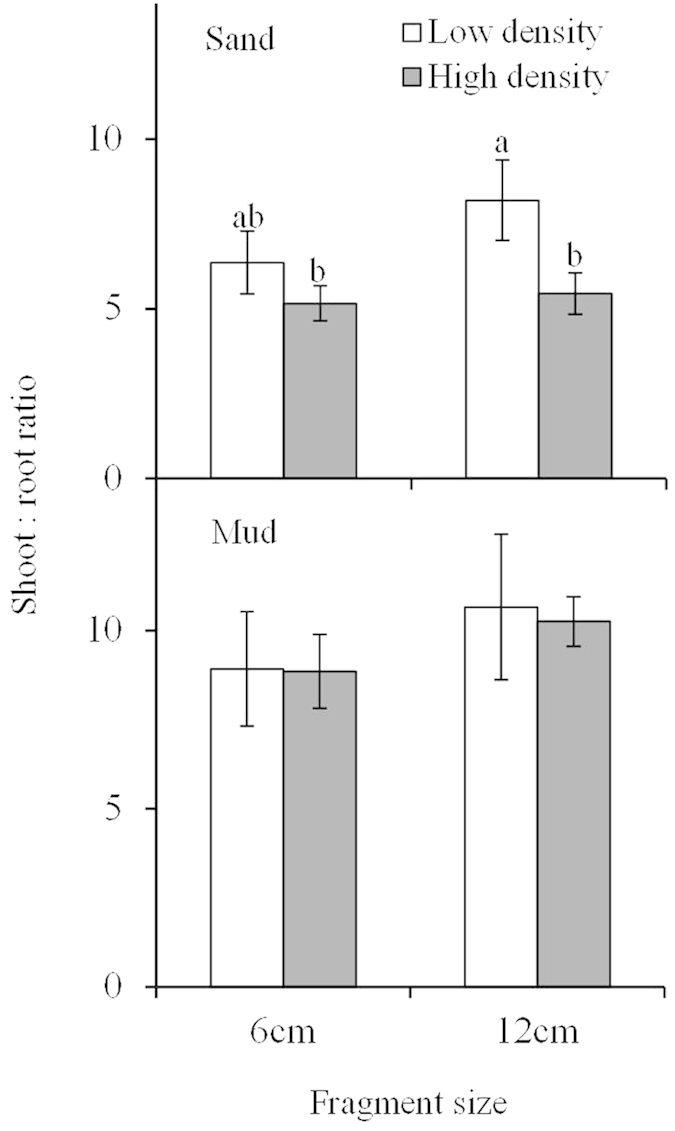
Biomass allocation (means ± SE, n = 5) of *Myriophyllum spicatum* with different fragment size growing in two densities and two sediment types.

The shoot:root ratio of *M. spicatum* was only significantly influenced by sediment type (Table 1; Fig. 3), which increased significantly in the mud treatment than that in the sand treatments (Fig. 3). The highest shoot:root ratio occurred in the low density +12 cm fragment + mud treatment (10.66 ± 2.04; Fig. 3), and was 2.07 times higher than the lowest shoot:root ratio in the high density +6 cm fragment + sand treatment (5.16 ± 0.52; Fig. 3).

### Total N and P content in the shoot and root

**Figure 4:**
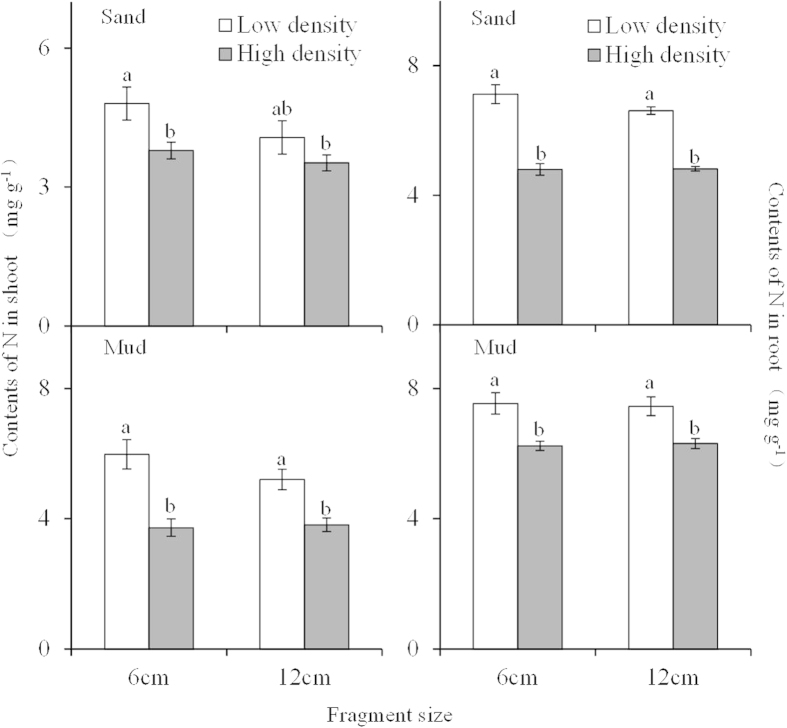
Total N contents (means ± SE, n = 5) in the shoot and root of *Myriophyllum spicatum* with different fragment size growing in two densities and two sediment types.

Total N content in the shoot and root was significantly affected by plant density and sediment type, but not fragment size (Tale 1; Fig. 4), and was much higher in the mud and low-density treatments than in the sand and high-density treatments. The highest shoot and root N content occurred in the low density + 6 cm fragment + mud treatment (5.98 mg g^−1^ and 7.54 mg g^−1^ in the shoot and root, respectively). These values were 1.70 and 1.57 times higher than the lowest values recorded for the high density +12 cm fragment + sand treatment and high density + 6 cm fragment + sand treatment for the shoot and root (3.52 mg g^−1^ and 4.80 mg g^−1^ in the shoot and root, respectively), respectively.

**Figure 5:**
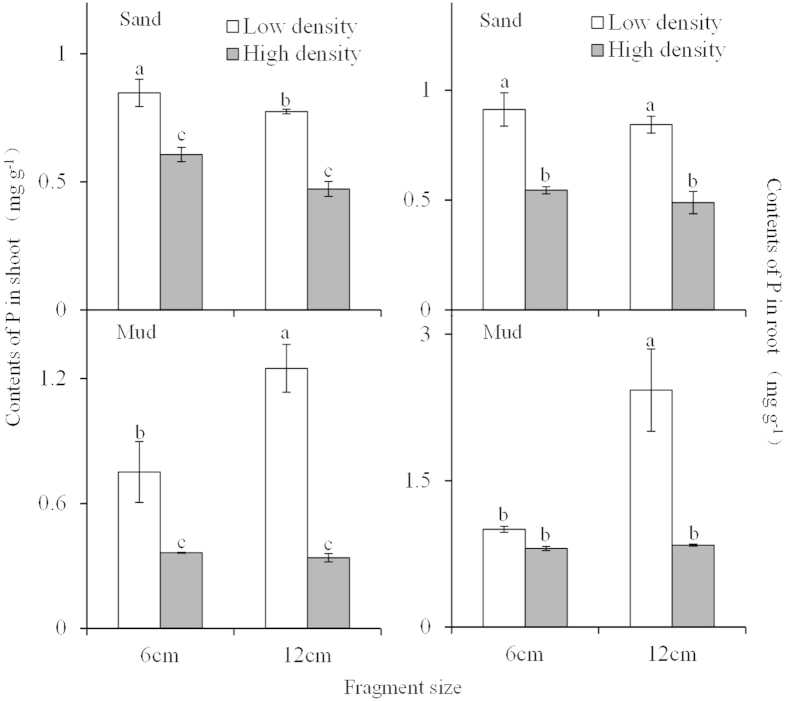
Total P contents (means ± SE, n = 5) in shoot and root of *Myriophyllum spicatum* with different fragment size growing in two densities and two sediment types.

Shoot P content only decreased with increasing plant density (Table 1; Fig. 5). In comparison, root P content was significantly influenced by sediment type, fragment size, and plant density (Table 1; Fig. 5), and was much higher in the mud and low-density treatments than in the sand and high-density treatments. Moreover, root P content in the low density +12 cm fragment + mud treatment was much higher than that in the low density + 6 cm fragment + mud treatment.

## Discussion

The RER, branching number and shoot diameter of *M. spicatum* were positively correlated with fragment size. These results were consistent with our hypothesis 1, indicating that larger fragments are favorable for the colonization of *M. spicatum.* Higher branching number in the 12 cm fragment indicates that larger fragments would produce more fragments, which would enhance its spread availability and, thus, increasing its geographical distribution^22^. Similar RGR for the two tested fragment sizes in all treatments indicated that this parameter had no significant influence on the growth of *M. spicatum*, which is in contrast to previous studies^8^. In general, previous studies have shown that smaller fragments have lower growth rates, possibly due to lower amounts of stored resources being available for new tissue production^6^,^8^. For instance, *M. spicatum* fragments of 5 cm length have higher growth rates than fragments of 2 cm length^8^. However, because the current study used fragment lengths >5 cm (i.e., 6 and 12 cm), the selected lengths might have been sufficient for *M. spicatum* to have enough resources for new tissue production. There was no significant difference in biomass allocation between the two fragment sizes in our experiment. One explanation is that the same resources were available for all fragments, particularly as the shoot : root ratio is negatively related to sediment nutrients^23^. Moreover, the results of total N content in the shoot and root and P content in the shoot (but not the root) indicated that nutrient content was similar for the different fragment size treatments.

Sediment provides the main nutrient resource for the growth of submerged macrophytes^18^, even though they may acquire nutrients via both the shoots and roots from the water and sediment, respectively^23^. In our experiment, RGR, RER, shoot diameter, and branching number exhibited a significant decrease in the sand treatments than in the mud treatments. These results are consistent with our first hypothesis and the results of previous studies^18^,^24^, indicating that the colonization ability of *M. spicatum* is significantly limited by sediment with low nutrient conditions. Lower N and P content in the shoot and root of *M. spicatum* in the sand treatments also reinforced this conclusion, supporting our third hypothesis. In addition, the lower shoot : root ratio in the sand treatment indicated that this species is able to allocate more energy to the root system to increase nutrient acquisition^25^. Furthermore, this species is able to adjust biomass allocation to different orders of roots (main roots, first-order lateral roots, and second-order lateral roots) and the structure of the root system in response to low nutrient conditions^20^. A field investigation of over 300 lakes in the USA reported that *M. spicatum* may cover 80% of lake area, even when the sediment composition is essentially 100% sand^26^, demonstrating that this species has high tolerance in various sediment types^18^.

In accordance with hypothesis 1, colonization ability was negatively correlated with plant density, which was reflected by lower RGR, RER, branching number, and shoot diameter in the high plant density treatment. The negative influence of plant density on plant growth has been widely studied^13^. For instance, *Potamogeton perfoliatus* produces more branches at lower densities^27^. In another study, the plant height of *Atriplex prostrata* decreased with increasing plant density^28^. In the sand sediment, a lower shoot : root ratio in the high-density treatment indicated that nutrient supply is limited, which was confirmed by the total N and P content in the shoot and root. Yet, this result contradicts previous studies, which found that the shoot : root ratio increased with increasing plant density^13^,^27^,^29^. In fact, at high plant densities, the pattern of biomass allocation might change depending on the resource that is in the most demand^30^,^31^. When nutrient availability meets the needs of plant growth, aboveground biomass is expected to increase, because the plant should shift more resources towards light-gathering structures. However, when nutrient availability cannot cover plant demand, the belowground biomass would be expected to increase^31^.

Our study also confirmed that the colonization ability was affected by fragment size, plant density, and sediment type, both independently and by their interactions (Table 1). For instance, in the sand treatment, the RGR of *M. spicatum* remained similar for both fragment sizes and plant density treatments, but decreased significantly with increasing plant density in the mud treatment. This result indicates that the influence of plant density is amplified by sediments with greater nutrient content, supporting the results of previous studies^13^,^32^. Higher nutrient availability facilitates plant growth, decreasing the growth limitation created by higher plant density. Elucidating this mechanism helps to improve our understanding about the response of fragment colonization to sediment types and plant densities.

Recently, the expansion of *M. spicatum* in lakes has caused many ecological problems in North America, North Africa, and China^18^,^33^,^34^. Here, we confirmed that larger fragment size, higher nutrient availability, and lower plant density facilitate *M. spicatum* colonization. This result demonstrates the mechanisms that regulate the expansion of this species, from which control measures could be developed. In addition to these three critical factors, other environmental factors (such as light and water levels, and interspecific competition) also influence the success of macrophyte fragment colonization^6^,^35^. Therefore, more studies are required to fully understand the factors that regulate *M. spicatum* colonization.

## Additional Information

**How to cite this article**: Li, F. *et al.* Colonization by fragments of the submerged macrophyte *Myriophyllum spicatum* under different sediment type and density conditions. *Sci. Rep.* **5**, 11821; doi: 10.1038/srep11821 (2015).

## Figures and Tables

**Table 1:** Summary of three – way ANOVAs for relative growth rate, relative elongation rate, biomass allocation, branching number, shoot diameter, total N content, and total P content in the shoot and root of *Myriophyllum spicatum* with different fragment size growing in two densities and two sediment types (F-values).

Variables	n	Sediment type (S)	Fragment size (F)	Density (D)	S×F	S×D	D×F	S×D×F
Relative growth rate (g g^−1^ day^−1^)	5	25.087***	0.239^NS^	24.534***	2.402^NS^	11.062**	2.411^NS^	1.212^NS^
Relative elongation rate (cm cm^−1^ day^−1^)	5	25.537***	354.728***	75.666***	1.688 ^NS^	7.121**	0.028 ^NS^	2.167 ^NS^
Branching number	5	33.312***	5.094*	58.906***	2.862^NS^	30.888***	6.524*	2.457 ^NS^
Shoot diameter (cm)	5	24.008***	9.072**	33.353***	0.533^NS^	14.233**	0.332^NS^	1.201 ^NS^
Shoot : root mass	5	16.413***	2.488^NS^	1.754^NS^	0.092^NS^	1.093^NS^	0.317^NS^	0.132^NS^
Shoot N (mg g^−1^)	5	8.859**	3.895^NS^	37.056***	0.133^NS^	5.901*	2.387^NS^	0.209^NS^
Root N (mg g^−1^)	5	52.896***	0.746^NS^	128.43***	0.677^NS^	8.327**	1.393^NS^	0.405^NS^
Shoot P (mg g^−1^)	5	0.001^NS^	1.791^NS^	85.588***	11.694**	14.370***	8.578**	5.390*
Root P (mg g^−1^)	5	25.165***	8.587**	30.294***	12.108**	5.434*	9.190**	9.514**

^NS^P > 0.05; *P < 0.05; **P < 0.01; ***P < 0.001.
